# The genus *Dasyproctus* (Hymenoptera, Apoidea, Crabronidae) in China, with description of two new species

**DOI:** 10.3897/zookeys.1025.59920

**Published:** 2021-03-18

**Authors:** Dan Yue, Li Ma, Qiang Li

**Affiliations:** 1 Department of Entomology, College of Plant Protection, Yunnan Agricultural University, Kunming, Yunnan, 650201, China Yunnan Agricultural University Kunming China

**Keywords:** Crabroninae, taxonomy, key, new species, new records

## Abstract

Two new species of the genus *Dasyproctus* Lepeletier de Saint Fargeau & Brullé (Crabronidae, Crabroninae, Crabronini) from China are described and illustrated, namely *D. amplicarinalis* Yue & Ma, **sp. nov.** from Yunnan, and *D. hainanensis* Yue & Li, **sp. nov.** from Hainan. In addition, *D. cevirus* Leclercq and *D. vaporus* Leclercq are recorded for the first time from China. A key to the species of *Dasyproctus* from China is provided.

## Introduction

The genus *Dasyproctus* Lepeletier de Saint Fargeau & Brullé, 1834 belongs to the subtribe Crabronina, tribe Crabronini, subfamily Crabroninae (Hymenoptera, Crabronidae). At present, *Dasyproctus* includes 79 species with 21 subspecies of small- to medium-sized predatory solitary wasps worldwide, of which 25 species and two subspecies occur in the Oriental Region, 37 species and 16 subspecies in the Afrotropical Region, 12 species in the Australasian Region, one species and one subspecies in both the Palearctic and Oriental Regions, two species in both the Palearctic and Afrotropical Regions, one species and two subspecies in both the Oriental and Afrotropical Regions, one species in both the Oriental and Australasian Regions, and one species in the Palearctic, Oriental, and Australasian Regions (Pulawski 2020; Binoy et al. 2021). In China, three species and one subspecies have been recorded from Foo Chow, Guangzhou, Szechwan, and Taiwan (Smith 1858; Cameron 1889, 1890; Turner 1912a, b; T. Ma 1936; Leclercq 1954, 1956, 1972, 1982, 2015; Tsuneki 1959, 1966, 1968, 1971, 1977, 1982; Haneda 1971, 1972; Murota 1973; Porter et al. 1999; Hua 2006).

In the present study of the *Dasyproctus* of China, two new species are described and two species are reported from China for the first time. A key to the Chinese species of the genus is provided.

## Materials and methods

The specimens examined during this study are deposited in the Insect Collection of Yunnan Agricultural University, Kunming, China (**YNAU**).

All specimens were observed and illustrated with the aid of an Olympus stereomicroscope (SZ Series) with an ocular micrometer. The photographs were taken with a Keyence VHX-5000. The final illustrations were improved for contrast and brightness using Adobe Photoshop CS5.

Terminology follows Bohart and Menke (1976). The abbreviations used in the text are as follows: **HL**, head length in dorsal view (distance from frons to occipital carina in the middle); **HW**, head width (dorsal view, maximum); **POD**, postocellar distance (distance between inner margins of hind ocelli); **OOD**, ocellocular distance (distance between outer margin of hind ocellus and nearest inner orbit). Size of punctures: small or fine, puncture diameters less than 0.1× posterior ocellar diameter; midsize, 0.1–0.2× posterior ocellar diameter.

## Systematics

### Dasyproctus

Taxon classificationAnimaliaHymenopteraCrabronidae

Lepeletier de Saint Fargeau & Brullé, 1834

186B4BA5-ABAE-5044-AC24-8469ABC9D178

Dasyproctus Lepeletier de Saint Fargeau & Brullé, 1834: 801. Type species: Dasyproctus bipunctatus Lepeletier de Saint Fargeau & Brullé, 1834, by monotypy.
Megapodium Dahlbom, 1844: 295. Type species: Megapodium westermanni Dahlbom, 1844, designated by Pate (1937: 37).
Bishamonis Tsuneki, 1983: 17, as subgenus of Dasyproctus. Type species: Dasyproctus guadalensis Tsuneki, 1983, by original designation and monotypy.

#### Diagnosis.

Body opaque or dull; scapal basin concave, simple or delimited dorsally by a carina; orbital fovea distinct to evanescent; antennal sockets contiguous with each other and with inner orbits; scape bicarinate; male flagellum simple or modified, most species without ventral setal fringe (except *D. araboides*); mandible bidentate apically in male, tridentate in female; pronotal collar with anterior carina reaching pronotal lobe in males and most females; postspiracular carina, omaulus, and acetabular carina present, contiguous; verticaulus elongate, sometimes inconspicuous; propodeum moderately sculptured, dorsal face micro-ridged, rugose, or areolate, enclosure not or inconspicuously defined, lateral propodeal carina well developed; legs simple or with hind femur modified; recurrent vein joining submarginal cell beyond its middle; jugal lobe shorter than submedian cell; gaster with first segment elongate-pedunculate; male without pygidial plate, female pygidial plate markedly narrowed, concave (Bohart and Menke 1976; Leclercq 2015).

### Dasyproctus amplicarinalis

Taxon classificationAnimaliaHymenopteraCrabronidae

Yue & Ma sp. nov.

8538FB99-7E4E-59ED-800F-5FBA3739ED4D

http://zoobank.org/F046D4FA-88E6-48F9-B299-3E03CABC4518

Figure 1a–g

#### Material examined.

***Holotype*.** ♀, China: Yunnan: Dehong: Yingjiang: Yunyan Mountain, 24°69'N, 97°93'E, 2005.VIII.15, coll. Li Ma (YNAU); ***Paratypes*.** 1♀, same place and date as holotype, coll. Kai Wu (YNAU); 1♀, China: Yunnan: Nujiang: Lushui, 25°97'N, 98°82'E, 2006.VII.19, coll. Li Ma (YNAU).

#### Diagnosis.

**Figure 1.**
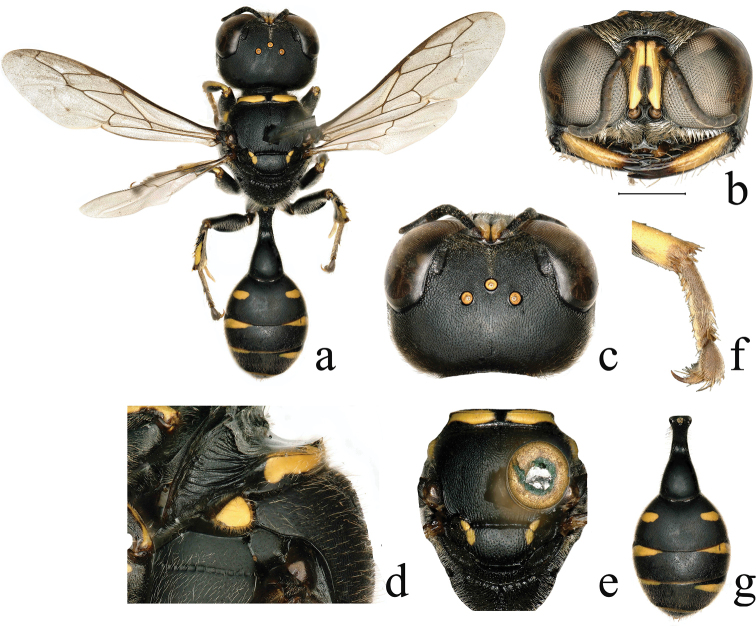
*Dasyproctus amplicarinalis* Yue & Ma, sp. nov., ♀. **a** habitus, dorsal view **b** head, frontal view **c** head, dorsal view **d** collar, lateral view **e** mesosoma, dorsal view **f** fore tarsomere I, dorsal view **g** metasoma, dorsal view. Scale bars: 0.43 mm (**e**); 0.49 mm (**a**); 0.57 mm (**g**); 1 mm (**b, c**); 1.31 mm (**f**); 1.48 mm (**d**).

The new species clearly differs from the Oriental *D. buddha* (Cameron) by the following combination of characters: frontal area dorsally with a high, lamellar, transverse carina at upper margin of scapal basin, carina interrupted by a broad and deep depression medially, and markedly high on each side of depression (Fig. 1c); antennal scape (except above with two black spots medially) yellow, pedicel brown (Fig. 1b); fore femur with one yellow spot; spots of tergum II larger than those of scutellum and tergum V (Fig. 1e, g). In *D. buddha*: frontal area dorsally with a lamellar, transverse carina at upper margin of scapal basin, carina interrupted by a narrow and shallow depression medially, and slightly higher on each side of depression; scape and pedicel yellow; fore femur with two separated yellow spots; spots of tergum II smaller than those of scutellum and tergum V.

The new species and *D. buddha* can be distinguished from the other species of the genus by the following combination of characters: free margin of clypeus truncate, slightly emarginated medially, lateral area with a blunt tooth on each side; frontal area dorsally with a lamellar, transverse carina at upper margin of scapal basin, carina interrupted by depression medially; anterior carina of pronotal collar laterally not curving toward pronotal lobe; length of petiole more than 2× maximum width.

#### Description.

**Female** (Fig. 1a). ***Body*** length 9.5–11.0 mm. Black; mandible largely pale yellowish (Fig. 1b); yellow are: scape (except above with two black spots) (Fig. 1b), pronotal collar (except black depression medially), pronotal lobe, anterior corner of scutellum, axilla (Fig. 1e), spot on ventral surface of fore femur subbasally, widely L-shaped band on mid femur ventrally (spot divided into two parts in some specimens), fore and mid tibiae ventrally and subapical 2/3 of hind tibia ventrally, spots on gastral terga II–V (Fig. 1g). Integument mostly with sparse, silvery setae; upper frons mostly and vertex entirely with sparse, golden setae; upper frons near transverse carina and frontal line with denser, golden setae; gena with short, dense, silvery appressed setae; scapal basin (except frontal line) with short, dense, golden setae; clypeus with dense, appressed, silvery setae; scape with white setae apically; lateral surface of mesosoma with dense, golden setae; gastral terga I–V with sparse, brown setae; sternum II with silvery setae and a nearly round setal spot laterally; posterior margin of sterna II–V with long, sparse, brown setae; tergum V laterally and pygidial plate basally and laterally with long, brown setae.

***Head*.** Mandible tridentate apically, inner side of mandible produced subapically; free margin of clypeus truncate, slightly emarginated medially, lateral area with a blunt tooth on each side (Fig. 1b); relative lengths of scape:pedicel:flagellum I:flagellum II:flagellum III = 100:18:29:22:20; frontal area dorsally with a high, lamellar, transverse carina at upper margin of scapal basin, carina interrupted by a broad and deep depression medially, and markedly high on each side of depression (Fig. 1c); orbital fovea shiny, oval, distinct, and large, length ca. 3× width, widest area slightly broader than hind ocellus diameter (Fig. 1c); upper frons with line formed by punctures (Fig. 1c), and with dense, small punctures 0.0–0.5× diameters apart; vertex with dense, small punctures ca. 1.5–2.0× diameters apart; gena with small punctures ca. 2–5× diameters apart; vertex to anterior ocellus with extremely fine midline (Fig. 1c). HL:HW:POD:OOD = 40:67:11:13.

***Mesosoma*.** Anterior carina of pronotal collar curving backwards in middle, laterally not curving toward pronotal lobe, nearly parallel to anterior margin of scutum, extending to insertion of fore coxa (Fig. 1d); pronotal collar with mid furrow (Fig. 1e); scutum with dense, midsize punctures ca. 1.0–1.5× diameters apart, and short, longitudinal rugae posteriorly; scutellum with dense, shallow, midsize punctures ca. 3–4× diameters apart and short, oblique longitudinal rugae posteriorly; mesopleuron with dense, shallow, midsize punctures ca. 1–4× diameters apart; metanotum with longitudinal rugae mixed with dense, midsize punctures ca. 0.8–1.0× diameters apart; metapleuron with coarse, oblique rugae; propodeal enclosure with oblique rugae and mid furrow; posterior surface with oblique rugae and mid furrow; lateral surface with dense, oblique rugae; outer margin of fore tarsomere I with three spines subbasally.

***Metasoma*.** Length of petiole 2.27× maximum width (Fig. 1g), and ca. half of hind femur, its surface with dense, midsize punctures ca. 1.5–2.0× diameters apart; terga with dense, fine punctures; sternum II with dense, small punctures; sterna III–VI posteriorly with dense, small to midsize punctures; terga II–V with yellow spot on each side, spots on terga III–IV larger than those of terga II and V, and spots on tergum II larger than those of tergum V (Fig. 1g); spots on tergum II larger than those of scutellum, but shorter than half of those of tergum III (Fig. 1e, g). Pygidial plate concave and narrow, with sparse, small to midsize punctures anteriorly, and with contiguous, midsize punctures and longitudinal rugae posteriorly.

**Male.** Unknown.

#### Distribution.

China (Yunnan).

#### Etymology.

The species’ name, *amplicarinalis*, is derived from the Latin stem *ampl*- (= large, spacious, roomy) and the Latin word *carinalis*, referring to the high, lamellar, transverse carina at the upper margin of the scapal basin, which is one of the main diagnostic characters of this species.

### Dasyproctus hainanensis

Taxon classificationAnimaliaHymenopteraCrabronidae

Yue & Li sp. nov.

510D3C19-3FA1-536F-95C2-8701E9FB1626

http://zoobank.org/62B586B8-1FF3-45AB-9F4C-860816D317BA

Figure 2a–g

#### Material examined.

***Holotype*.** ♀, China: Hainan, 18°10'–20°10'N, 108°37'–111°05'E, 1934.VIII.2, coll. Qi He (YNAU).

#### Diagnosis.

**Figure 2.**
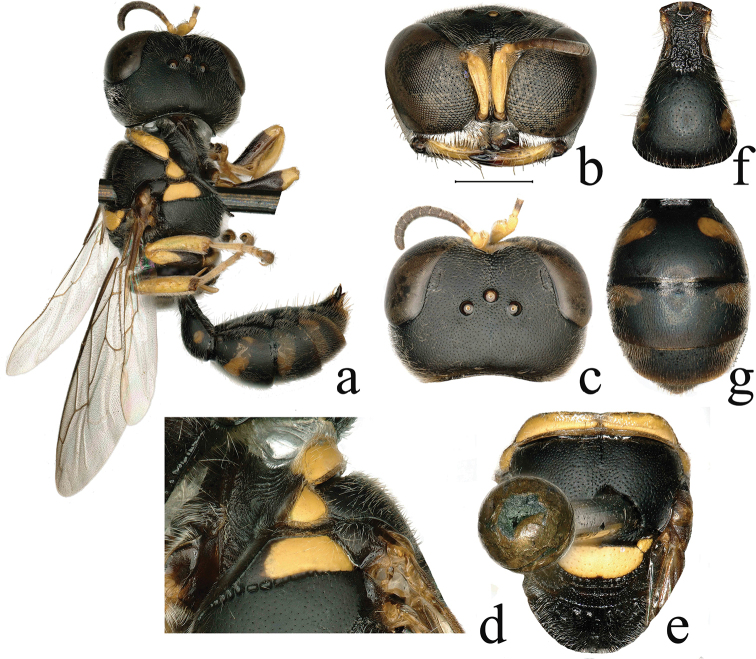
*Dasyproctus hainanensis* Yue & Li, sp. nov., ♀ **a** habitus, lateral view **b** head, frontal view **c** head, dorsal view **d** collar, lateral view **e** mesosoma, dorsal view **f** petiole, gastral tergum I, dorsal view **g** gastral terga II–V, dorsal view. Scale bars: 0.68 mm (**a**); 0.89 mm (**e, g**); 0.99 mm (**c**); 1 mm (**b**); 1.31 mm (**f**); 1.65 mm (**d**).

The new species clearly differs from the Oriental *D. pentheri* Leclercq by the following combination of characters: free margin of clypeus with a deep, triangular emargination medially, laterally with an angular tooth on each side (Fig. 2b); propodeal enclosure with sparse, sturdy, longitudinal rugae; tibiae largely yellow (inner surface brown). In *D. pentheri*, the free margin of the clypeus has a deep semicircular emargination medially, the lateral area has a round tooth on each side; the propodeal enclosure has irregular, slender rugae; the tibiae are largely black.

The new species and *D. pentheri* can be distinguished from the other species of the genus by the following combination of characters: free margin of clypeus with a deep emargination medially, lateral area with a tooth on each side; anterior carina of pronotal collar laterally curving toward pronotal lobe; length of petiole no more than 2× maximum width (Fig. 2f).

#### Description.

**Female** (Fig. 2a). ***Body*** length 7.8 mm. Black; yellow are: mandible (largely), two spots on clypeus medially, scape, pedicel, and flagellomere I subbasally, pronotal collar, pronotal lobe, prepectus (largely), scutellum, axilla, outer and inner sides of fore femur apically, apical half of mid femur, fore and mid tibiae, outer surface of hind tibia; fore coxa apically, inner sides of fore and mid trochanters, and tarsus pale yellowish; spots on gastral terga I–II laterally yellowish-brown, spots on terga III–IV laterally ferruginous, bands on tergum V and posterior margin of terga IV–V ferruginous (Fig. 2f, g). Integument mostly with sparse, silvery setae; clypeus with dense appressed silvery setae; scapal basin (except frontal line) with short, dense, appressed, silvery setae; upper frons and vertex with sparse, pale brown setae; gena with short, somewhat dense, silvery setae; gastral terga largely with sparse, brown setae; gastral sterna II–V with long, somewhat sparse, brown setae; setae on sternum II irregular, setae on sterna III–V in nearly linear arrangement, sternum II laterally with oval setal spot; pygidial plate anterolaterally with long, brown setae.

***Head*.** Mandible tridentate apically; median lobe of clypeus with mid carina, free margin with deep, triangular emargination medially, lateral area with an angular tooth on each side (Fig. 2b); relative lengths of scape:pedicel:flagellum I:flagellum II:flagellum III = 35:7:8:9:6; frontal area dorsally with an inconspicuous, transverse carina at upper margin of scapal basin (Fig. 2c); orbital fovea shiny, distinct, large, oval, length ca. 3× width, widest area slightly narrower than hind ocellus diameter (Fig. 2c); upper frons with line formed by punctures, and with somewhat dense, small to midsize punctures ca. 0.0–1.5× diameters apart; gena with dense, fine punctures ca. 1–2× diameters apart; vertex with somewhat dense, small punctures ca. 2× diameters apart; vertex to anterior ocellus with extremely fine midline (Fig. 2c). HL:HW:POD:OOD = 8:6:2:3.

***Mesosoma*.** Anterior carina of pronotal collar laterally curving toward pronotal lobe (Fig. 2d), pronotal collar with mid furrow (Fig. 2e); scutum with somewhat dense, small punctures ca. 1–3× diameters apart, posterior margin with sparse, small punctures ca. 2–5× diameters apart and short, oblique rugae; axilla with sparse, shallow, midsize punctures ca. 0.0–1.5× diameters apart; scutellum (middle area impunctate) with sparse, shallow, midsize punctures ca. 4.5–5.5× diameters apart and short, longitudinal rugae posteriorly; posterior area shiny, alutaceous, and with dense, midsize punctures, precoxal sulcus with sparse, small punctures anteriorly, metanotum with coarse, longitudinal carinae mixed with sparse, coarse punctures; metapleuron with coarse, oblique rugae; propodeal enclosure with sparse, coarse, longitudinal rugae and mid furrow, lateral area with sparse, oblique rugae; posterior surface with dense, transverse rugae, and narrow, deep mid furrow; lateral surface with dense, fine, oblique rugae.

***Metasoma*.** Length of petiole 1.22× maximum width (Fig. 2f), and half of hind femur, its surface rough, with contiguous large punctures; gastral terga I–IV with a yellowish-brown or ferruginous spot on each side, tergum V with a band, spots on tergum I slightest of all (Fig. 2g); gastral terga with dense, fine punctures, gastral sterna with sparse, small to midsize punctures; pygidial plate concave and narrow, posteriorly with contiguous, small to midsize punctures and longitudinal rugae.

**Male.** Unknown.

#### Distribution.

China (Hainan).

#### Etymology.

The new species is named after the Hainan Province of China, where the holotype was collected.

### Dasyproctus cevirus

Taxon classificationAnimaliaHymenopteraCrabronidae

Leclercq, 1963, first record from China

D801BBBE-816D-56F6-B4F4-70940FDEA3E7

Figure 3a–g

Dasyproctus cevirus Leclercq, 1963: 16.

#### Material examined.

1♀, China: Hainan: Baisha, 2008.IV.29, coll. Chengjin Yan; 1♀, China: Hainan: Bangxi Polu Nature Reserve, 2008.V.3, coll. Chengjin Yan; 1♀, China: Hainan: Lanyang Park, 2002.VII.18, coll. Zaifu Xu; 1♀, China: Hainan: Songtao Reservoir, 2002.VII.17, coll. Zaifu Xu; 1♀, China: Yunnan: Anning, 2006.VII.7, coll. Ming Luo; 2♀, China: Yunnan: Baoshan: Longyang District, 2006.VII.18, coll. Rui Zhang; 2♀, China: Yunnan: Baoshan: Longyang: Lu Jiang Ba Thermal Institute, 2006.VII.20, coll. Rui Zhang; 5♀4♂, China: Yunnan: Dehong: Luanchuan, 2005.VIII.13, coll. Chunju Liu (3♀2♂), Tingjing Li (1♀), Kai Wu (1♀2♂); 4♀2♂, China: Yunnan: Dehong: Luxi, 2005.VIII.9–10, coll. Li Ma (2♀2♂), Tingjing Li (1♀), Xiaoli Li (1♀); 1♀2♂, China: Yunnan: Dehong: Ruili, 2005.VIII.12, coll. Tingjing Li (1♂), Li Ma (1♀1♂); 9♀2♂, China: Yunnan: Dehong: Yingjiang, 2005.VIII.15–16, coll. Kai Wu (1♀1♂), Chunju Liu (1♀), Tingjing Li (4♀), Li Ma (3♀1♂); 2♀, China: Yunnan: Hekou, 2003.VII.21, coll. Zhenshan Geng (1♀), Qiang Li (1♀); 1♀, China: Yunnan: Kunming: Songhua dam, 2006.VII.29, coll. Wenliang Li; 2♂, China: Yunnan: Lincang: Linxiang, 2004.X.5, coll. Li Ma (1♂), Chunju Liu (1♂); 1♀, China: Yunnan: Lijiang: Ninglang, 2005.VIII.25, coll. Tingjing Li; 2♂, China: Yunnan: Mengla: Wangtianshu Forest Park, 2005.V.2, coll. Peng Wang; 3♀1♂, China: Yunnan: Mengla, 2005.V.8, coll. Peng Wang; 2♀, China: Yunnan: Mengla, 2005.V.20–21, coll. Peng Wang; 3♀, China: Yunnan: Nujiang: Fugong, 2003.VIII.24, coll. Peng Wang; 3♀, China: Yunnan: Nujiang: Lishui, 2006.VII.19, coll. Li Ma (1♀), Rui Zhang (2♀); 6♀3♂, China: Yunnan: Simao: Jingdong, 2005.IV.28–V.1, coll. Chunju Liu (2♂), Baoxin Dong (3♀), Kai Wu (1♀1♂), Hesheng Wang (2♀); 13♀4♂, China: Yunnan: Simao: Jinggu, 2004.X.4, coll. Baoxin Dong (2♀), Chunju Liu (1♀), Kai Wu (1♀1♂), Li Ma (7♀2♂), Hesheng Wang (1♀1♂), Haiyan Zhang (1♀); 1♀, China: Yunnan: Kunming: Yunnan Agricultural University, 2007.VIII.15, coll. Peng Wang; 1♀, China: Yunnan: Xishuangbanna: Jinghong: Daluo Forest Park, 2004.X.2, coll. Kai Wu; 1♀, China: Yunnan: Jinghong, 2004.X.3, coll. Baoxin Dong (all YNAU).

#### Distribution.

China (Hainan, Yunnan); Philippines; Thailand; Indonesia; Papua New Guinea.

**Figure 3.**
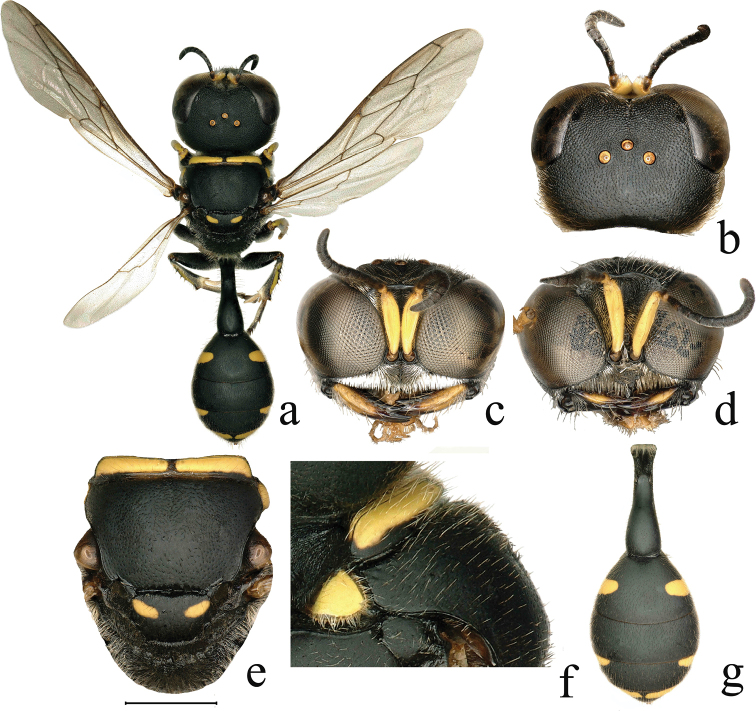
*Dasyproctus cevirus* Leclercq **a** ♀. habitus, dorsal view **b** ♀. head, dorsal view **c** ♀. head, frontal view **d** ♂. head, frontal view **e** ♀. mesosoma, dorsal view **f** ♀. collar, lateral view **g** ♀. metasoma, dorsal view. Scale bars: 0.47 mm (**a**); 0.62 mm (**g**); 0.81 mm (**b**); 0.85 mm (**c**); 1 mm (**e**); 1.09 mm (**d**); 1.79 mm (**f**).

### Dasyproctus vaporus

Taxon classificationAnimaliaHymenopteraCrabronidae

Leclercq, 1963, first record from China

0A953216-5E40-595F-8A03-0C352027CECF

Figure 4a–f

Dasyproctus vaporus Leclercq, 1963: 22.
Dasyproctus sculpturatus Tsuneki, 1976: 113, synonymized with Dasyproctus vaporus by Tsuneki (1984: 16).

#### Material examined.

1♂, China: Yunnan: Dehong: Luxi, 2005.VIII.10, coll. Tingjing Li; 5♂, China: Yunnan: Dehong: Yingjiang: Yunyan Mountain, 2005.VIII.15, coll. Li Ma (2♂), Haiyan Zhang (2♂), Tingjing Li (1♂); 1♂, China: Yunnan: Dehong: Yingjiang, 2005.VIII.15, coll. Kai Wu; 1♂, China: Yunnan: Dehong: Yingjiang: Taiping Township, 2005.VIII.15, coll. Xiaoli Li (all YNAU).

#### Distribution.

China (Yunnan); Philippines.

**Figure 4.**
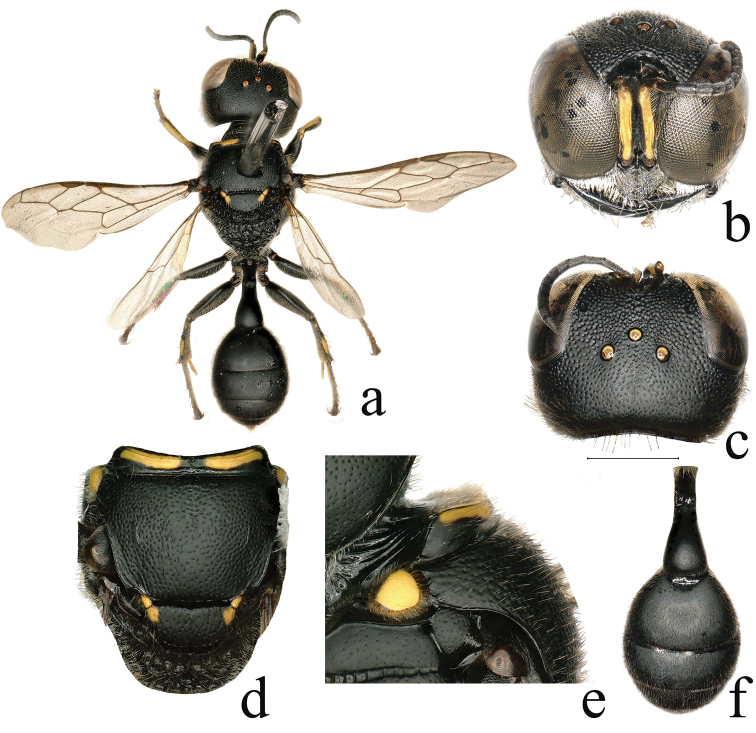
*Dasyproctus vaporus* Leclercq, ♂ **a** habitus, dorsal view **b** head, frontal view **c** head, dorsal view **d** mesosoma, dorsal view **e** collar, lateral view **f** metasoma, dorsal view. Scale bars: 0.46 mm (**a**); 0.67 mm (**f**); 1 mm (**b, c**); 1.07 mm (**d**); 1.64 mm (**e**).

### Key to the species of *Dasyproctus* from China

Females

**Table:** 

1	Anterior carina of pronotal collar laterally not curving toward pronotal lobe (Fig. 1d)	**2**
–	Anterior carina of pronotal collar laterally curving toward pronotal lobe (Figs 2d, 3f)	**4**
2	Gastral tergum II with basal depression; free margin of clypeus with broad triangular concavity	***D. jungi* T. Ma**
–	Gastral tergum II without basal depression; free margin of clypeus nearly truncate, slightly emarginated medially	**3**
3	Frontal area dorsally with a high, lamellar, transverse carina at upper margin of scapal basin, carina interrupted by a broad and deep depression medially, and markedly high on each side of depression (Fig. 1c); scape yellow (except above with two black spots medially), pedicel brown; outer margin of fore femur with one yellow spot	***D. amplicarinalis* sp. nov.**
–	Frontal area dorsally with a lamellar, transverse carina at upper margin of scapal basin, carina interrupted by a narrow and shallow depression medially, and slightly higher on each side of depression; scape and pedicel yellow; fore femur with two yellow spots	***D. buddha* (Cameron)**
4	Petiole broad and short, length not exceeding twice its maximum width, and about half of hind femur (Fig. 2f)	***D. hainanensis* sp. nov.**
–	Petiole longer, length of petiole more than twice its maximum width, and longer than hind femur	**5**
5	Free margin of clypeus with deeply semicircular, median emargination (Fig. 3c); carina at anterior margin of pronotal collar more or less minutely produced near middle of each half (Fig. 3e); hind basitarsus apically brown	***D. cevirus* Leclercq**
–	Free margin of clypeus with deeply triangular or semicircular emargination medially; carina at anterior margin of pronotal collar not produced anteriorly, either straight or gently incurved and sinuate; hind basitarsus largely brown	**6**
6	Clypeus with golden pubescence; metapleuron with fine, transverse striation	***D. agilis orientalis* (Cameron)**
–	Clypeus with silvery pubescence; metapleuron with conspicuous, close, oblique striation basally, remainder with fine, close, longitudinal striation	***D. agilis agilis* (F. Smith)**

Males

**Table:** 

1	Gastral tergum II with basal depression; gastral terga without yellow spots (rarely present) or bands	***D. jungi* T. Ma**
–	Gastral tergum II without basal depression; gastral terga with yellow spots or bands	**2**
2	Free margin of clypeus with angular projection medially	**3**
–	Free margin of clypeus without angular projection, variable medially	**4**
3	Free margin of clypeus more sharply pointed medially (Fig. 3d); outer surface of hind tibia black	***D. cevirus* Leclercq**
–	Free margin of clypeus broad and blunt medially; outer surface of hind tibia mostly yellow	***D. agilis agilis* (F. Smith)**
4	Free margin of clypeus slightly emarginated medially, lateral area without a tooth on each side; gastral terga II and IV with a yellow spot on each side (rarely missing), tergum III without a spot, tergum V or terga V–VI with a broad yellow band	***D. agilis orientalis* (Cameron)**
–	Free margin of clypeus truncate or slightly emarginated medially, lateral area with a tooth on each side; gastral terga II and IV with a yellow spot on each side or a yellow band, tergum III with a spot on each side, terga V and VI with a yellow band, or rarely with a small spot on each side	**5**
5	Free margin of clypeus truncated medially, lateral teeth at acute angle to middle lobe; scutum with fine punctures, usually smaller than punctures on vertex; gastral tergum III with a pair of spots, spots distinctly larger than those on tergum IV (rarely missing) and tergum II (rarely present), terga V and VI rarely with a small spot on each side	***D. buddha* (Cameron)**
–	Free margin of clypeus slightly emarginated medially, lateral teeth at 90° to middle lobe (Fig. 4b); scutum with relatively large punctures, similar to punctures on vertex; gastral terga II–III with a spot on each side (missing in some specimens), terga IV–VI with a broad yellow band (missing in some specimens) (Fig. 4f)	***D. vaporus* Leclercq**

## Supplementary Material

XML Treatment for Dasyproctus

XML Treatment for Dasyproctus amplicarinalis

XML Treatment for Dasyproctus hainanensis

XML Treatment for Dasyproctus cevirus

XML Treatment for Dasyproctus vaporus
